# Diquafosol tetrasodium elicits total cholesterol release from rabbit meibomian gland cells via P2Y_2_ purinergic receptor signalling

**DOI:** 10.1038/s41598-021-86433-6

**Published:** 2021-03-26

**Authors:** Ken-ichi Endo, Asuka Sakamoto, Koushi Fujisawa

**Affiliations:** Pharmaceutics and Pharmacology, Research and Development Division, Santen Pharmaceutical Co., Ltd., 8916-16 Takayama-cho, Ikoma, Nara 630-0101 Japan

**Keywords:** Cell signalling, Biochemistry, Cell biology, Physiology

## Abstract

Diquafosol tetrasodium (DQS), a purinergic P2Y_2_ receptor agonist, stimulates secretion of both water and mucins from the conjunctiva into tears. Hence, DQS-containing eye drops have been approved as a therapeutic option for dry eye disease in some Asian countries, including Japan. Recent clinical reports state that instilling DQS-containing eye drops significantly increases the lipid layer thickness in tears. Therefore, we examined this compound’s direct actions on holocrine lipid-secreting meibomian gland cells and their function. Isolated meibomian gland cells (meibocytes) were procured from rabbits and cultivated in serum-free culture medium. Differentiated meibocytes with pioglitazone were used for the subsequent experiments. Intracellular Ca^2+^ signalling of the cells was dramatically elevated with DQS addition in a dose-dependent manner. This DQS-induced elevation was almost completely cancelled by the coexistence of the selective P2Y_2_ receptor antagonist AR-C118925XX. DQS treatment also facilitated total cholesterol (TC) release from cells into the medium. This effect of DQS on TC was suppressed significantly by the intracellular Ca^2+^ chelator BAPTA-AM as well as by AR-C118925XX. DNA fragmentation analysis revealed that DQS may have enhanced the apoptotic DNA fragmentation caused spontaneously by cells. Thus, DQS could stimulate meibocytes to release lipids through the P2Y_2_ receptor and possibly facilitate holocrine cell maturation.

## Introduction

Dry eye disease has been established as “a multifactorial disease of the ocular surface, characterized by a loss of homeostasis of the tear film, and accompanied by ocular symptoms, in which tear film instability and hyperosmolarity, ocular surface inflammation and damage and neurosensory abnormalities play etiological roles” and as “a multifactorial disease, characterized by unstable tear film, causing a variety of symptoms and/or visual impairment, potentially accompanied by ocular surface damage” by the Tear Film and Ocular Surface Society Dry Eye Workshop II and the Asia Dry Eye Society, respectively^1,2^. Tear film instability and the importance of visual dysfunction associated with this disease, and provision of insufficient components in each tear film layer and ocular surface epithelium, are gaining acceptance as fundamental therapeutic concepts^3,4^.

The ocular surface is composed of two layers, aqueous-mucin and lipid layer. The aqueous fluid of tears is produced by the lacrimal gland as the major source. Secretory and membrane-associated mucins, derived from the corneal and conjunctival epithelia, contribute to tear film stabilisation by creating the appropriate interface to the ocular surface.

Diquafosol tetrasodium (DQS), a uridine 5′-triphosphate (UTP) analogue, is an agonist of the purinergic P2Y_2_ receptor. This receptor is a membrane protein, encoded by the *P2RY2* gene, and belongs to the family of G-protein coupled receptors. It responds to extracellular purine and pyrimidine nucleotides, especially UTP and adenosine 5′-triphosphate (ATP), to promote diverse cellular physiological functions by eliciting Ca^2+^ release from the endoplasmic reticulum. P2Y_2_ receptors are found at several anterior eye sites, such as the corneal and conjunctival epithelia and conjunctival goblet cells, and DQS stimulates the secretion of both water and mucins from cells into tears^5,6^. Because of these pharmacological actions, ophthalmic solutions containing DQS have been approved as therapeutic options for dry eye disease in some Asian countries, including Japan, South Korea and China.

Besides water and mucins, lipids that form the outermost layer of the tear film are believed to be important in stabilising tear film since they help prevent water evaporation from tears. Most lipids in tears, such as wax esters, triglycerides and total cholesterol (TC), i.e., the sum of cholesterol esters and free cholesterol, are supplied as meibum from the meibomian glands, located in the upper and lower eyelids^7^. Meibomian glands as same as sebaceous glands grow from ectoderm sheets, but the process of development is different. The basic characteristics of the meibomian glands have some parts in common with the sebaceous glands, but there are also some differences in anatomy, secretory regulation, composition of lipids. It is known that meibocyte finally secretes meibum by a holocrine mode after maturation accompanied by intracellular lipid production and accumulation^8^. Recently, several clinical studies^9–11^ have reported that the lipid layer in tears thickened significantly after instilling DQS eye drops in patients with dry eyes and healthy volunteers. The meibomian gland epithelial cells (meibocytes) also express the P2Y_2_ receptor^12^. We explored whether DQS acted directly on the meibocytes, as well as on the conjunctival cells, and their physiological function.

## Results

### Primary culture of rabbit meibocytes

Inoculated cells were cultured to form colonies under serum-free condition. The cells exhibited a cobblestone-like appearance, similar to that reported in previous research^13^ (Supplementary Fig. S1a,c). Cell differentiation was induced using pioglitazone, after which the cells exhibited numerous lipid droplet-like particles, visualised by Nile red fluorescent staining, similar to that in the tissue (Fig. S1b). Without the PPAR agonist, the cells were stained diffusely in the cytoplasm with the Nile red dye (Fig. S1d).

### *P2RY2* mRNA expression in cultured meibocytes

As reported previously, the *P2RY2* gene was expressed in the meibomian gland epithelium of monkey by the in situ hybridisation method^12^. We evaluated *P2RY2* gene expression in rabbit meibocytes. Quantitative RT-PCR results demonstrated that, just after harvesting, rabbit meibocytes certainly expressed the *P2RY2* transcript, with a delta cycle threshold (ΔCt) of 10.1. The *P2RY2* transcript was also detected at a similar level (134% of just after harvesting [ΔCt = 9.7]) in meibocytes cultured with the PPAR agonist. These results indicated that rabbit meibocytes, both in vivo and in our culture system, expressed a relative level of the *P2RY2* gene transcript. Based on the gene expression and lipid staining results, we used the cells differentiated by the agonist for all experiments in this study.

### Increase in intracellular Ca^2+^ in meibocytes by DQS

**Figure 1:**
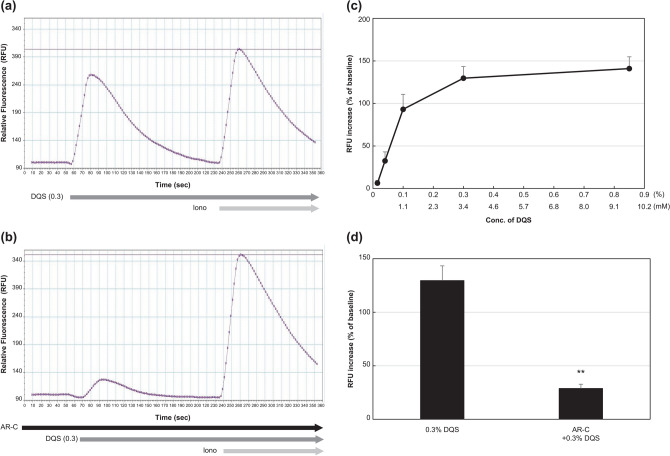
Effect of DQS on intracellular Ca^2+^ signalling of cultured meibocytes. (**a**) Representative fluorescence chart of DQS (0.3%) addition following ionomycin (0.5 µM). (**b**) The DQS chart, following ionomycin, under coexistence of AR-C118925XX (3 µM). (**c**) Integrated result of the increase in intracellular Ca^2+^ signalling by DQS. (**d**) Integrated result of the suppression of intracellular Ca^2+^ signalling by AR-C118925XX (3 µM) in DQS-treated meibocytes. Each value represents mean ± S.E.M. obtained from four independent experiments. **p < 0.01, compared with DQS (0.3%).

As depicted in Fig. 1a,c, intracellular Ca^2+^ signalling in the culture increased dramatically after DQS addition (from 0.015% to 0.85%) in a dose-dependent manner. Under the coexistence of AR-C118925XX (3 µM), this increase was almost completely suppressed (p = 0.004), whereas the response to ionomycin (0.5 µM) remained unchanged (Fig. 1b,d). This implied that the purinergic P2Y_2_ receptor signalling action was present in the meibocytes. The EC_50_ of DQS was calculated as 0.078% ± 0.011% (approximately 0.9 mM) in this assay, which was much higher than that of other reports using different cells^14,15^. We speculated that ectonucleotidases in the meibocyte culture degraded DQS rapidly. In fact, the EC_50_ of UTPγS, an enzymatically stable analogue of UTP, was approximately 3.3 µM in this assay.

### DQS stimulated the release of TC from meibocytes

**Figure 2:**
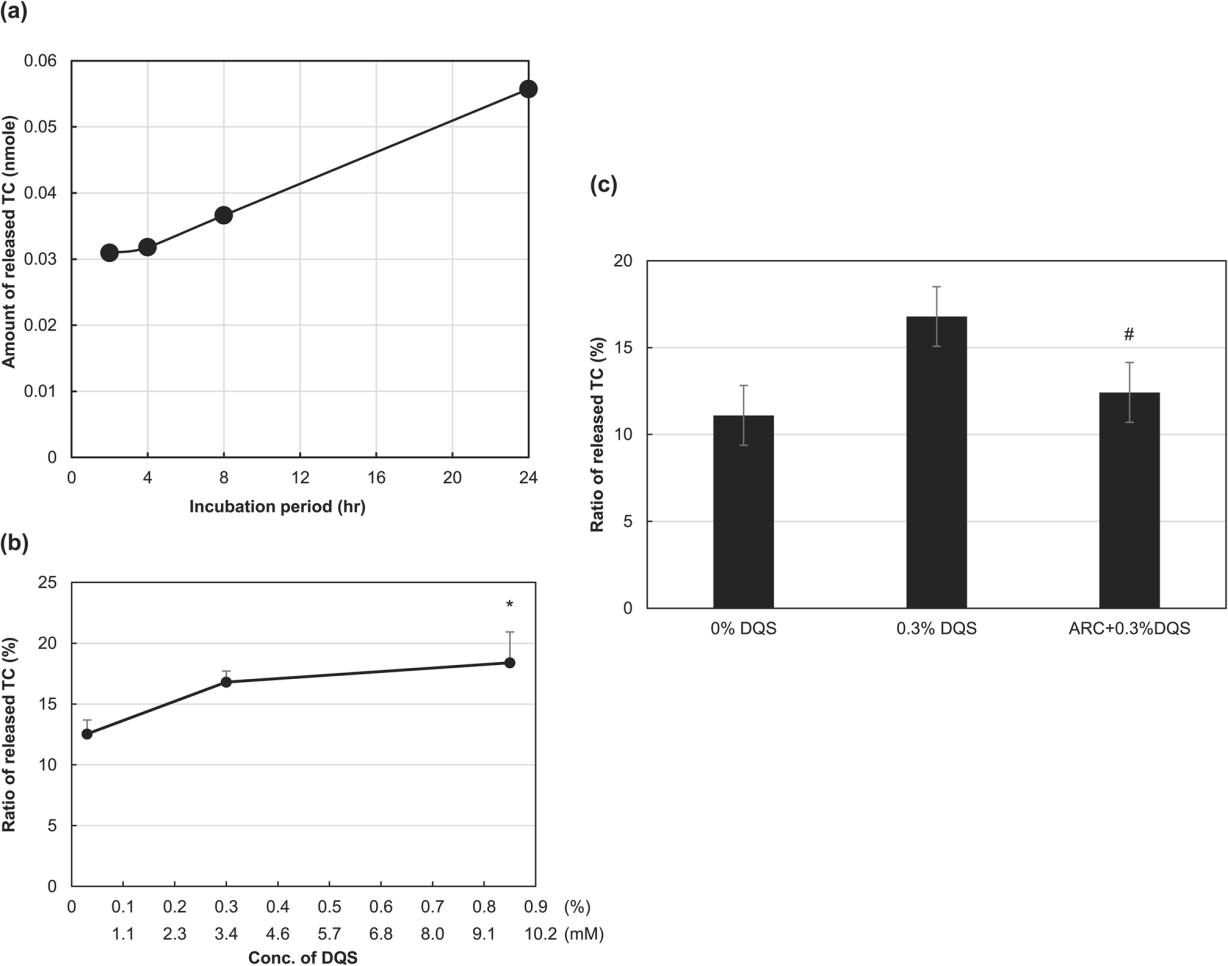
Effect of DQS on released ratio of TC from cultured meibocytes. (**a**) TC accumulation in the culture media during incubation without DQS. (**b**) Integrated result on the released ratio of TC by DQS. (**c**) Integrated result of the suppression of released TC by AR-C118925XX (3 µM) in DQS-treated meibocytes. Each value represents mean ± S.E.M. obtained from 5 independent experiments. *p < 0.05, compared with control, ^#^p < 0.05 compared with DQS (0.3%).

To determine whether P2Y_2_ receptor signalling led to lipid secretion in meibocytes, the TC release from the cells was examined. TC released from the cell into the culture medium accumulated gradually over the incubation period (Fig. 2a), indicating that the cells in the culture continuously secreted lipid molecules. Based on preliminary study results, we adopted 4 h as the incubation period in the present experiment. Like the intracellular Ca^2+^ signalling, the released ratio of TC increased significantly with DQS concentration in a dose-dependent manner (p = 0.024 with 0.85% DQS) (Fig. 2b). AR-C118925XX considerably (p = 0.045) suppressed the TC increase induced by 0.3% DQS (Fig. 2c). The intracellular Ca^2+^ chelator BAPTA-AM (3 µM) also suppressed the increase considerably (Supplementary Fig. S2). These results indicated that increased intracellular Ca^2+^ signalling, through the P2Y_2_ receptor signalling, enhanced lipid secretion in cells.

### Apoptotic DNA ladder formation in DQS-treated meibocytes

**Figure 3:**
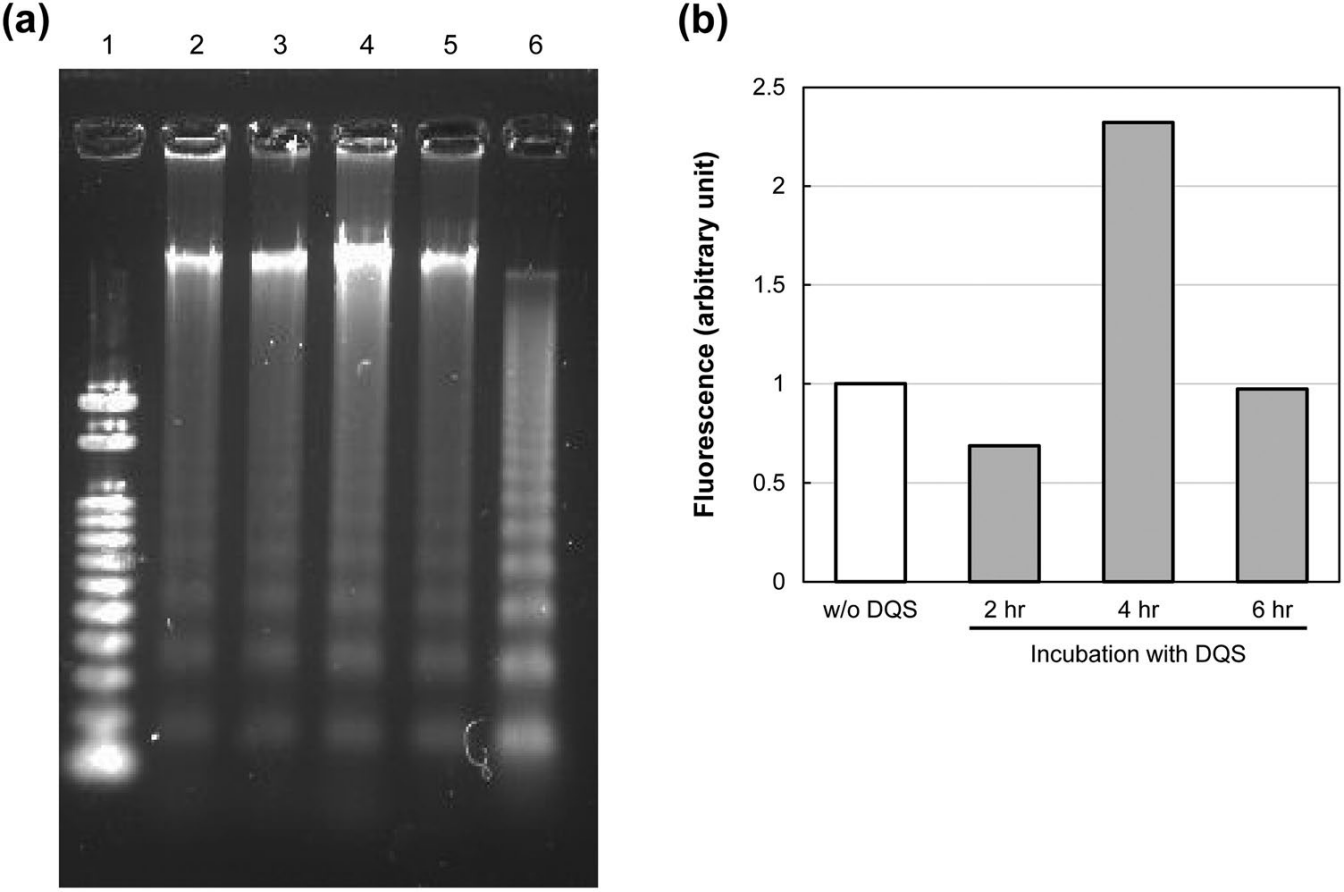
Analysis of DNA fragments isolated from meibocytes treated with DQS. (**a**) Representative image of agarose gel electrophoresis. Lane 1, DNA size marker (100 bp); lane 2, without DQS; lanes 3–5, treated with DQS (0.85%) for 2, 4 and 6 h, respectively; lane 6, positive apoptotic control. (**b**) Quantification of the DNA fragments. Each intensity of fluorescence is represented as ratio of control (w/o DQS).

In holocrine cells, secretion is always accompanied by programmed cell death, such as apoptosis. In fact, apoptotic (TUNEL-positive) cells in the acini were observed in the meibomian gland around the openings facing the duct (Supplementary Fig. S3). We examined apoptotic DNA ladder formation in DQS-treated meibocytes, which was detected even in the DQS-untreated cells (Fig. 3a and Supplementary Fig. S4), indicating that some cells in our culture system exhibited spontaneous apoptotic DNA fragmentation. Although the DNA ladder patterns of DQS-treated cells appeared indistinguishable from those of control, the quantitation analysis revealed that DQS treatment facilitated DNA fragment formation in the meibocytes at a maximum of 4 h (Fig. 3b).

## Discussion

This study demonstrated that DQS elicited intracellular Ca^2+^ increase and TC release in rabbit meibocytes in vitro. In addition, the results obtained by treatment with the selective P2Y_2_ receptor antagonist AR-C118925XX against these cellular responses argue that these DQS actions are undoubtedly mediated through the purinergic P2Y_2_ receptor signalling. Kam et al.^16^ used immortalised human meibomian gland epithelial cells and reported that 100 µM UTP had no effect on neutral lipid contents, including free cholesterol, in the cells. Our results clearly demonstrated that the effective concentration of DQS in the meibocyte culture was far higher (i.e., EC_50_ in Ca^2+^ flux = 0.9 mM) than that used by those authors, supposedly due to non-negligible ectonucleotidases. Hence, they might need to use the agonist at higher concentrations or use an enzymatically stable agonist, such as UTPγS, instead of UTP. Meibum that is secreted by meibomian glands contains various lipid species, such as wax esters, triglycerides, free fatty acids and phospholipids, besides TC. Concerning lipid profile of meibocytes, it is reported that several phospholipids (> 50%) and cholesterol (26–28%) are dominated in human meibomian gland epithelial cell line (HMGEC). Wax esters is one of the main lipids in meibum, however it is considerably few (< 1%) in HMGEC^17^. We focused on TC in this study because it was a major lipid in meibocytes and its available commercial test kit had adequate sensitivity. Nevertheless, it might be necessary to evaluate other lipid species in the future and specific difference in lipid profile of meibocytes.

Although several variations of physiological cell death, including apoptosis, autophagy, pyroptosis, ferroptosis, cornification and necroptosis, have been discovered^18–20^, the mode of cell death associated within the holocrine process and the mechanism of holocrine remain unidentified, even in sebum-secreting cells (sebocytes). Recently, Fischer et al.^21^ examined epithelial cell-specific DNase-ablated mouse lines and demonstrated that holocrine secretion in sebaceous glands was driven by a unique lysosomal DNase2-mediated programmed cell death, which differed from apoptosis, cornification and necroptosis. Regarding the meibomian gland, a previous study (Jester JV, et al. IOVS 2016;57:ARVO E-Abstract 5661) suggested that holocrine secretion involves both apoptotic and autophagic pathways, based on the immunochemical locations of Caspase 3a and 9, Beclin 1 and ULK1 proteins in this tissue. Our results of TUNEL staining and DNA ladder formation indicated that some fully differentiated meibocytes meet their fate with apoptosis-like properties. The intracellular Ca^2+^ signalling, induced by DQS, appears to facilitate, rather than disturb, the cells to undergo their physiological cell fate. Indeed, it is well characterized that the increase in intracellular Ca^2+^ concentration is key trigger event undergoing to programmed cell death like apoptosis^22^. It is known that higher concentrations of ATP or UTP induce apoptosis in some cancer cell lines^23–25^. However, DQS should not be considered a cytotoxic agent because DQS (3%) decreases reactive oxygen species generation and inhibits apoptosis in dry-conditioned human corneal epithelial cells^26^.

As mentioned previously, this study was conducted based on recent clinical studies on tear lipid layer thickness after instilling commercially available ophthalmic solutions containing DQS. However, our findings concerning the effect of DQS on meibocyte functions might not account for the clinical evidence provided by those previous studies because the onset of the effect of the ophthalmic solution on the tear lipid layer seems extremely rapid (within 20 min) to induce holocrine secretion accompanied by apoptosis-like cell death. In other words, DQS could facilitate both lipid secretion and meibum delivery from the ducts to the lid margin reservoir. Arita et al.^27^, who analysed projected meibography images quantitatively, reported that the projected area of meibomian glands increased dramatically in all patients with obstructive meibomian gland dysfunction after more than four months of DQS therapy, suggesting that topical DQS activated some existing meibomian glands to produce meibomian lipid. We found in this study that DQS directly stimulated a holocrine-like lipid secretion on meibocytes, suggesting that DQS facilitates the turnover and differentiation of meibocytes.

There are some limitations in this study. First, diquafosol actually causes holocrine-like TC release from meibocytes, and TC is thought to derive from cells that have undergone apoptosis, but there is insufficient direct evidence between apoptosis and TC secretion. Second, it is not yet clear whether the effects of diquafosol on meibocytes observed in this study have led to actual clinical effects of this drug. Our greatest interest, for the present, is whether it penetrates in meibomian glands to achieve more than effective concentration for the cells by instillation of available eye drops. Further studies are required to address this issue.

In conclusion, DQS could stimulate meibocytes to release lipids through the P2Y_2_ receptor and possibly facilitate holocrine cell maturation.　Therefore, it is suggested that such an action of DQS on the lipids release from meibocytes may have a benefit in patients with evaporative-type dry eye accompanied by a decrease in tear lipid layer.

## Methods

### Reagents

Sterilised isotonic aqueous solution containing 8.5% DQS (pH 7.5) was supplied by our company’s formulation facility and diluted with basal Ham’s F-12 medium (Nacalai Tesque, Kyoto, Japan) before use. In addition, the following reagents were used in this study: recombinant human epidermal growth factor (hEGF; Corning, One Riverfront Plaza, NY); collagenase A, dispase II and O-phosphorylethanolamine (Millipore Sigma, St. Louise, MO); pioglitazone hydrochloride (Tokyo Chemical Industry, Tokyo, Japan); ITS-X (100 ×) and Nile red dye (Thermo Fisher Scientific, Waltham, MA); Ca^2+^ ionophore ionomycin calcium (FUJIFILM Wako Pure Chemical, Osaka, Japan); selective P2Y_2_ receptor antagonist AR-C118925XX (Bio-Techne, Minneapolis, MN) and intracellular Ca^2+^ chelator BAPTA-AM (Abcam, Cambridge, UK); and PrimeGel Agarose PCR-Sieve (Takara Bio, Shiga, Japan). The other products and reagent kits used in this study are described in the experimental procedures.

### Experimental animals

Male New Zealand White and Japan White rabbits, weighing 2.5–3.5 kg, were obtained from Kitayama Labes (Nagano, Japan) for this study. All animal care and experimental procedures were performed according to the ARVO Statement for the Use of Animals in Ophthalmic and Vision Research and were approved and monitored by the Animal Care and Use Committee at Santen Pharmaceuticals. Approval Number and Date: DR-2019-0009 (8th Apr. 2019), DR-2019-0149 (11th Jul. 2019), DR-2019-0212 (10th Sep. 2019) and DR-2019–0425 (9th Mar. 2020). This study was also carried out in compliance with the ARRIVE guideline (http://www.nc3rs.org.uk/page.asp?id=1357).

### Primary culture of rabbit meibocytes

Rabbit meibocytes were isolated and cultured, essentially according to a previous report^13^ with minor modifications. Briefly, rabbits were instilled with a drop of levofloxacin ophthalmic solution (Cravit; Santen Pharmaceutical, Osaka, Japan) into both eyes and then sacrificed by applying an overdose intravenous injection of pentobarbital anaesthetic for animals (Somunopentil; Kyoritsu Seiyaku, Tokyo, Japan). After sterilising the eyelid skin and the surrounding area with diluted Isodine (Mundipharma, Tokyo, Japan), both the upper and lower eyelids were excised. The conjunctival mucosa was removed by curretting with an ophthalmic scalpel. Subsequently, the cutaneous segment of the eyelid and the tarsal muscle lamella were removed to the extent possible. The residual posterior lamella containing meibomian glands was cut into small pieces, parallel to the glandular units. These tissue pieces were digested in 0.5% collagenase A plus 0.6 U/mL dispase II at 37 °C for 2–3 h. Then, each glandular unit was carefully isolated under a binocular stereomicroscope and placed together. To dissociate them into cells, the tissues were immersed in 2 mM EDTA in PBS for 5 min, followed by trituration. Cells were suspended in a culture medium, consisting of Ham’s F-12-containing ITS-X (1 ×), 0.1 mM O-phosphorylethanolamine, 10 ng/mL hEGF, 0.1% DMSO and 50 µg/mL gentamycin, and inoculated at a density of 1.5 × 10^4^ cells/well into a clear-bottom 96-well black plate (BD Biosciences, MA, USA). The cultures were incubated at 37 °C under humidified 5% CO_2_ + 95% air phase. The next day, half the volume of culture supernatant was replaced with fresh culture medium. Thereafter, the medium change was performed 3 or 4 times weekly. To promote cell differentiation^28^, the cells were treated with 10 µM pioglitazone, a peroxisome proliferator-activated receptor (PPAR) agonist, for 1–3 days before using them in all subsequent experiments.

Nile red staining was applied for observing intracellular lipids. The staining working solution was prepared by mixing 5 mL of Ham’s F-12, 2 µL of Nile red stock solution (0.1 mg/mL in acetone) and 5 µL of Cellstain Hoechst 33342 solution (Dojindo Laboratories, Kumamoto, Japan). After washing twice, the cultures were incubated with the staining working solution for 30 min at room temperature. Then, the cultures were again washed twice and observed under an inverted fluorescence microscope equipped with Hoffman’s modulation contrast system (Olympus, Tokyo, Japan).

### Quantitative RT-PCR for assessing *P2RY2* mRNA expression

Meibocytes that were just harvested and those cultured with the PPAR agonist were, respectively, lysed with Qiazol lysis reagent (Qiagen, Hilden, Germany), after which total RNA purification and cDNA synthesis were performed using RNeasy Universal Mini Kit (Qiagen) and PrimeScript RT Master Mix (Takara Bio), respectively, according to the manufacturers’ instructions. To quantify the *Oryctolagus cuniculus P2RY2* (accession No. XM_008264329) and *GAPDH* (NM_001082253) transcripts, the oligonucleotide pair of 5′-AGACGTGGGTGGTGTGAGC-3′ and 5′-GCCGTTCGCAGTGCCATTC-3′ and the pair of 5′-TTCAACAGTGCCACCCACTC-3′ and 5′-CGTTGTCATACCAGGAAATGAGC-3′ were, respectively, used as specific primer sets with the QuantiFast SYBR Green PCR Kit (Qiagen) according to the manufacturer’s instructions. PCR steps were performed in the 7500 Fast Real-Time PCR System (Thermo Fisher Scientific). Relative *P2RY2* expression was determined by the standard ΔΔCt method using *GAPDH* as housekeeping gene.

### Ca^2+^ mobilisation assay

For this assay, a combination of Screen Quest Calbryte-520 Probenecid-Free and Wash-Free Calcium Assay Kit (AAT Bioquest, Sunnyvale, CA) and the FlexStation3 fluorescence plate reader (Molecular Devices, San Jose, CA) was used. Cultures were incubated with equal volumes of the dye-loading solution at 37 °C for 50 min, followed by incubation at room temperature for 30 min with AR-C118925XX in some case. DQS and ionomycin were sequentially injected using the FlexStation3 system to achieve the desired final concentrations during sequential fluorescence reading.

### Measurement of TC contents

To calculate the released ratio of TC, cellular and extracellular TC levels were respectively measured using the Amplex Red Cholesterol Assay Kit (Thermo Fisher Scientific), following the protocol using cholesterol esterase described by the manufacturer. The culture supernatant from the cell cultures incubated in Ham’s F-12 containing DQS at 37 °C was collected, and then an equal volume of 1 × reaction buffer, provided in the kit, was added. To extract the lipid content from the cells, the residual cells were incubated with a lipid extraction solution [hexane:isopropanol = 3:2 (v/v)]^29^ for 30 min at room temperature, and then the extraction solution was collected into a glass vial. Lipid extraction was performed once again, and the pooled extract was dried at 70 °C. The cellular lipid extract was dissolved with the mixture of the aqueous reaction buffer and Ham’s F-12 (1:1). All samples were heated at 60 °C for 30 min to inactivate the enzymes that could interfere with the assay^30^.

### Isolation and detection of DNA fragments

Cells plated into the 6-well culture plate were incubated with DQS (0.85%) for 0–6 h and then collected using a cell scraper. DNA fragments in the cellular samples were isolated using the ApopLadder Ex Kit (Takara Bio), according to the manufacturer’s instructions. This kit allows selective extraction of small DNA fragments from apoptotic cells while minimising the contamination of intact chromatin DNA. The resultant DNA pellet was dissolved in 30 µL of Tris–EDTA (TE) buffer. To visualise the DNA ladder, an aliquot (10 µL) of the sample, mixed with 2 µL of 6 × loading buffer, was electrophoresed in 1.5% agarose gel with 1 × Tris-Acetare-EDTA (TAE) buffer. Then, the gel was stained with 1 × TAE containing SYBR Green I dye. Apoptotic control sample in the Apoptotic DNA Ladder Kit (Roche) was used as a positive control. Quantification of the DNA fragments was essentially performed as described in a previous report^31^ according to the ApopLadder Ex Kit instruction manual. Briefly, 20 µL of samples, diluted with TE buffer, was mixed with 2 µL of diluted (1:1,000) SYBR Green I dye. The fluorescence intensity (Ex: 485 nm, Em: 538 nm) was measured using the FlexStation3 plate reader.

### TdT-mediated dUTP nick end labelling (TUNEL) staining

The eyelid tissue of the sacrificed rabbit was fixed with neutralised buffered formalin, followed by embedding in paraffin. The deparaffinised tissue section was treated with proteinase K (Agilent, Santa Clara, CA) and then with 3% hydrogen peroxide. The TUNEL reaction was performed using components of the in situ Apoptosis Detection kit (Takara Bio). The tissue section was stained using Histofine DAB substrate kit (Nichirei Bioscience, Tokyo, Japan) and then with hematoxylin 3G (Sakura Finetek Japan, Tokyo, Japan).

### Statistical analysis

Four or five independent experiments were conducted for the Ca^2+^ mobilisation assay and TC assay. Data obtained from these experiments are expressed as mean ± S.E.M. Statistical analyses were performed using SAS-based EXSUS software (version 10.0.3; CAC Croit, Tokyo, Japan). Statistical differences were determined by a two-tailed *t*-test for two groups and the two-tailed Dunnett’s multiple comparison test for three or more groups. Statistical significance was p < 0.05.

### Ethics declarations

All animal care and experimental procedures were performed according to the ARVO Statement for the Use of Animals in Ophthalmic and Vision Research and were approved and monitored by the Animal Care and Use Committee at Santen Pharmaceuticals. DR-2019–0009 (8th Apr. 2019), DR-2019–0149 (11th Jul. 2019), DR-2019–0212 (10th Sep. 2019) and DR-2019–0425 (9th Mar. 2020).

## Supplementary Information

Supplementary Information.

## Data Availability

The data sets generated and analysed in the present study may be available from the corresponding author upon request.
