# Supplementary material for: Diquafosol tetrasodium elicits total cholesterol release from rabbit meibomian gland cells via P2Y2 purinergic receptor signalling
Source: Sci Rep. 2021 Mar 26;11:6989. doi: 10.1038/s41598-021-86433-6 (PMC7997929; doi:10.1038/s41598-021-86433-6)
Supplement: Supplementary file 1 — Supplementary Information. [file 41598_2021_86433_MOESM1_ESM.pdf]

Diquafosol sodium elicits total cholesterol release from rabbit meibomian gland cells via $P2Y_2$ purinergic receptor signalling

Ken-ichi Endo[1,*], Asuka Sakamoto[1], Koushi Fujisawa[1]

[1]Pharmaceutics & Pharmacology, Research and Development Division, Santen Pharmaceutical Co., Ltd., Nara, Japan.

*Corresponding author

Pharmaceutics & Pharmacology, R&D Division, Santen Pharmaceutical Co., Ltd.,

8916-16 Takayama-cho, Ikoma-shi, Nara 630-0101, Japan

E-mail: kenichi.endo@santen.com

Tel: +81-743-79-4525, Fax: +81-743-79-4591

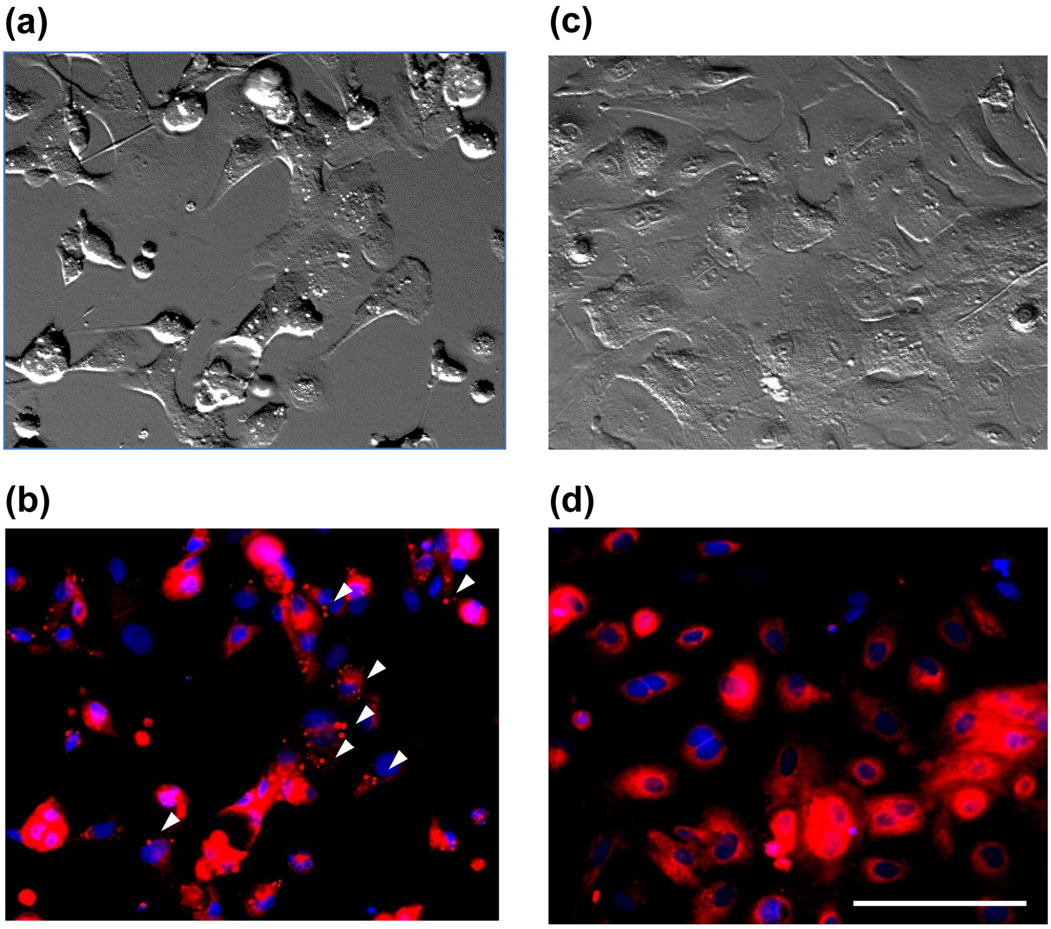

Supplementary Figure S1. Microscopic images of rabbit meibocyte culture after induction of differentiation

Cells were cultured in serum-free culture medium following treatment with (a, b) and without (c, d) 10 μM pioglitazone. (a, c) The bright fields with Hoffman's modulation contrast system. (b, d) The fluorescent images stained with Nile red (red) and Hoechst 33342 (blue). Arrowheads indicate droplet-like Nile red staining in the cells, bar = 150 μm.

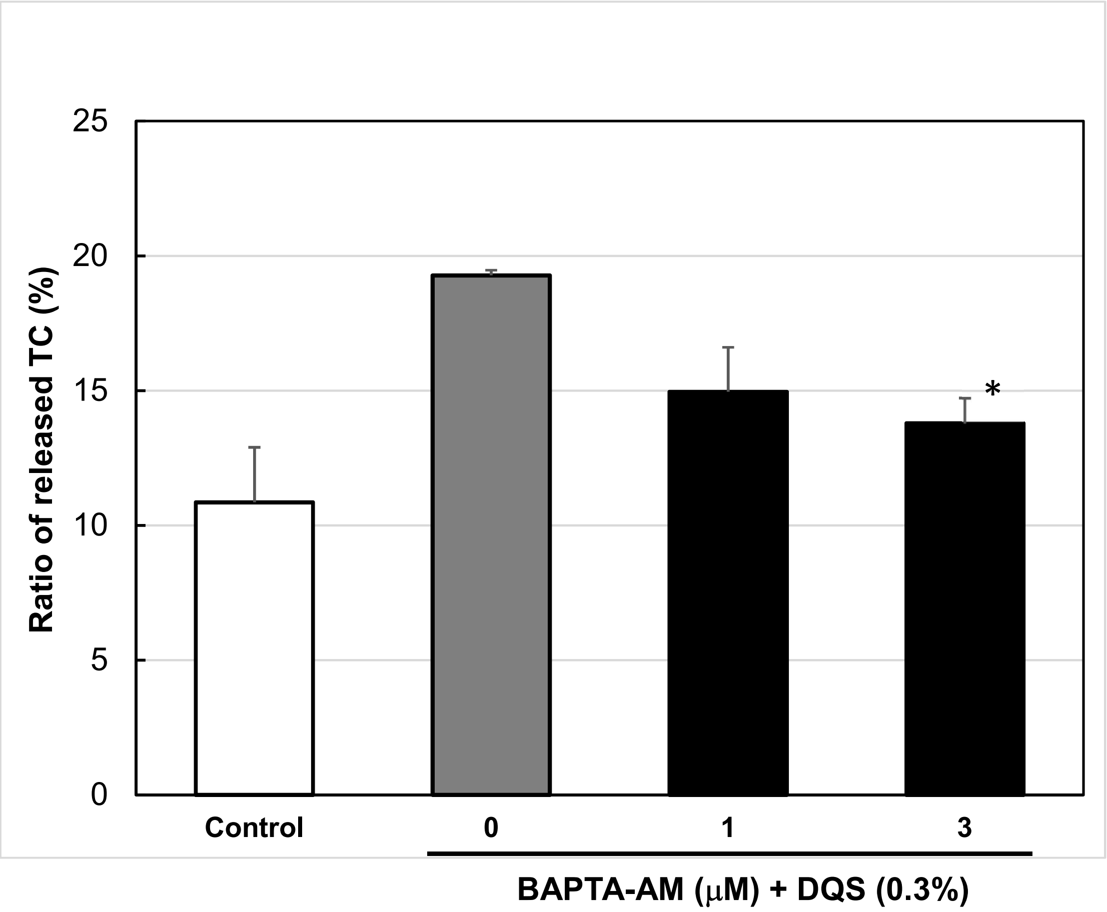

Supplementary Figure S2. Effect of BAPTA-AM on increase of TC release from the meibocytes caused by DQS

Each value represents mean ± S.E.M. obtained from 3 culture replicates. *$p < 0.05$, compared with DQS only.

(a) (b)

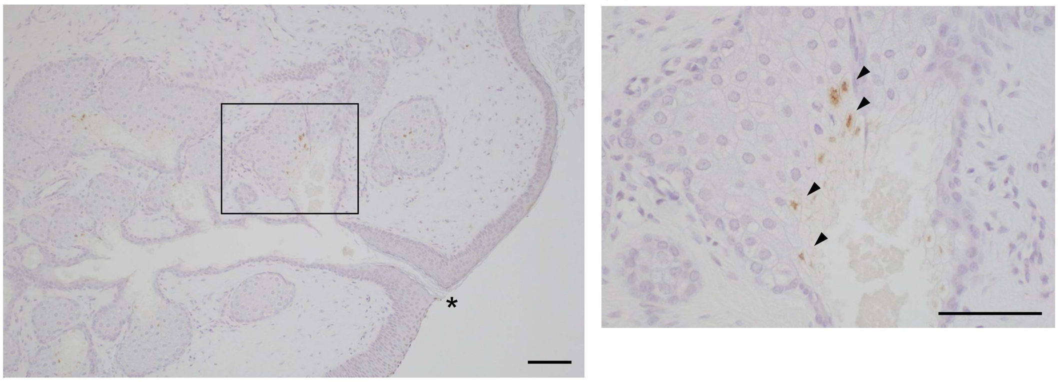

Supplementary Figure S3. TUNEL staining on histological section of rabbit eyelid (a) Longitudinal section along a unit of meibomian gland. (b) The boxed area in right image under high magnification. Asterisk (*) indicates the orifice of meibomian gland unit. Cells having brownish nucleic residues (arrowheads) indicate TUNEL-positive cells, bar = 100 μm.

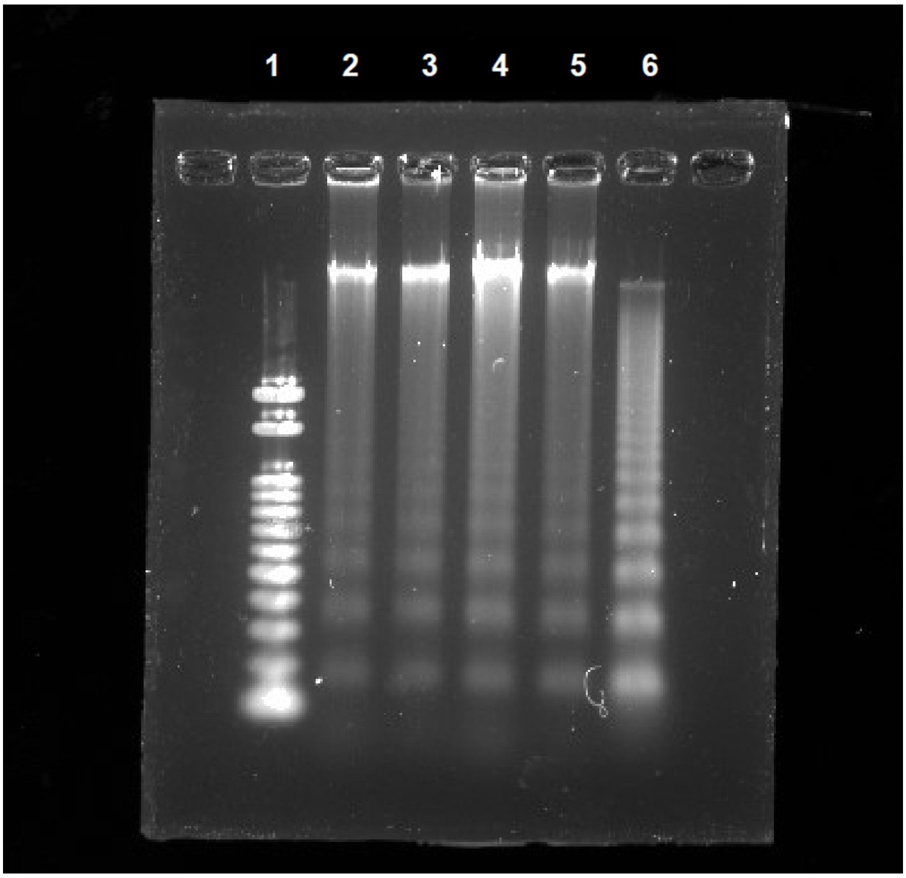

Supplementary Figure S4. Full-length image of the agarose gel electrophoresis displayed in Fig. 3a

See Figure 3 for the correspondence between lane numbers and samples.
